# Using patient storytelling to improve medical students’ empathy in Japan: a pre-post study

**DOI:** 10.1186/s12909-023-04054-1

**Published:** 2023-01-27

**Authors:** Yumi Kagawa, Hirono Ishikawa, Daisuke Son, Tsuyoshi Okuhara, Hiroko Okada, Haruka Ueno, Eiko Goto, Aiko Tsunezumi, Takahiro Kiuchi

**Affiliations:** 1 Department of Health Communication, School of Public Health, The University of Tokyo, 7-3-1 Hongo, Bunkyo-ku, Tokyo, 113-8655 Japan; 2 School of Public Health, Teikyo University, Tokyo, Japan; 3 Department of Community-based Family Medicine, Faculty of Medicine, Tottori University, Tottori, Japan; 4 Department of Health and Dietetics, Faculty of Health and Medical Science, Teikyo Heisei University, Tokyo, Japan

**Keywords:** Patient storytelling, Empathy, Perspective-taking, Patient-centered practice, Humanistic education, Jefferson Scale of Empathy-Student version, JSE-S, Undergraduate medical education, Japan

## Abstract

**Background:**

Empathy for patients is now internationally accepted as one of the competencies of physicians for patient-centered medical practice and an essential component of medical education. Recently, “patient storytelling” has attracted attention in empathy education for medical students to understand patients’ experiences, feelings, and perspectives. This study aimed to quantitatively evaluate how patient storytelling enhanced undergraduate medical students’ empathy in Japan to the extent that they sustained it for six months.

**Methods:**

Participants were 159 fourth-year undergraduate medical students in Tokyo in academic years 2018 and 2019. The questionnaire surveys were conducted three times: at the beginning of the class, immediately after the class, and six months after the class. The Japanese version of the Jefferson Scale of Empathy-Student Version was used in this study. Gender, age, and clinical orientation were also obtained through the self-reported questionnaire. We invited a male patient storyteller who was diagnosed with chronic kidney disease to the classes on “Professionalism.” The title of his storytelling was “The Power of Medical Professionals’ Words.”

**Results:**

JSE-S scores improved significantly immediately after listening to patient storytelling. The scores remained improved six months after the class. Interest of specialty was significantly positively associated with an immediate change in JSE-S scores. However, gender had no significant association with changes in JSE-S scores either immediately or six months after education.

**Conclusions:**

Our findings may suggest that patient storytelling would be useful to cultivate empathy among undergraduate medical students. It is to be expected that more medical schools will use patient storytelling to educate medical students in humanistic and communication education.

**Supplementary Information:**

The online version contains supplementary material available at 10.1186/s12909-023-04054-1.

## Background

Numerous studies have confirmed the importance of physicians’ empathy in several key aspects, such as improved patient satisfaction [1–3], greater adherence to therapy [2, 4], better clinical outcomes [5–8], and lower malpractice liability [9]. Empathy for patients is now internationally accepted as one of the competencies of physicians for patient-centered medical practice and an essential component of medical education.

In cognitive development theory, empathy is not only an emotional trait (vicarious emotional response) but also a primary cognitive ability (perspective-taking and empathic concern) [10]. One commonly accepted definition for a patient care situation is “a cognitive attribute that involves an ability to understand the patient’s inner experiences and perspective and a capability to communicate this understanding” [11].

Predictors of medical students’ empathy have been intensively studied. Gender difference in empathy has been attributed to intrinsic factors (e.g., evolutionary-biological gender characteristics) as well as extrinsic factors (e. g., interpersonal style in caring, socialization, and gender role expectation) [11]. Consistent with studies in the U.S., Japanese studies showed that female Japanese medical students achieved higher empathy scores than male medical students [12, 13]. Other studies reported that higher baseline empathy scores, Asian ethnicity, and students’ interest of specialty were associated with a greater increase in empathy. As for specialty, those who were interested in “people-oriented” specialty that required a higher level of empathic involvement obtained a significantly higher empathy score than those who were interested in pursuing “skills- or procedure-oriented” specialties [14–16]. For these reasons, there is a need for an educational strategy to help male medical students as well as “skills/procedure-oriented” medical students understand empathy and make it effective in their clinical practice.

According to adult learning theory, which evolved from cognitive development theory, the development of perspective-taking skills is facilitated by experiential and reflective learning [17]. Therefore, empathy education is highly compatible with patient narrative, which allows learners to relive the experiences, feelings, and thoughts of characters differently from the narrators through narrative information [18–20]. Previous studies have attempted to use patient narratives in medical education, including videos of patient interviews [21, 22], documentary films [23], and patient memoirs [24]. Qualitative studies have reported that this type of education is useful for medical students in developing a patient’s perspective of the pain, emotions, and lifeworld [25–27]. However, quantitative studies are scarce, and even in studies that have reported improvements in empathy scores, the effect size has been small [21–24].

Recently, “patient storytelling” has attracted attention in empathy education for medical students [27–29]. Patient storytelling is more realistic and interactive than teacher readings or videos. Therefore, patient storytelling is expected to have a different educational effect on medical students’ understanding of patients’ perspectives from that of previous studies. In fact, no previous studies have quantitatively examined the educational effect of patient storytelling on medical students’ empathy for patients. Additionally, most of the previous studies on medical students’ empathy, including education using narratives, have reported their effect immediately after the education, but few of them have examined even the medium-to long-term sustainability of the educational effect [23, 30]. There is also a lack of studies examining the improvement of empathy and its associated factors in medical students through education using patient storytelling.

In Japan, the medical education curriculum released in 2016 added a new section on professionalism and the academic goal of understanding patient values and supporting patient self-determination [31]. However, evidence-based educational methods that effectively encourage medical students to understand the patient perspective are still lacking in Japan [32].

This study aimed to quantitatively evaluate the extent to which education in listening to patient storytelling improved medical students’ empathy for patients. Specifically, we set three research questions (RQ) as follows:

1. To what extent did medical students’ empathy for patients improve immediately after listening to the patient storytelling?
2. After six months of listening to the patient storytelling, did they sustain the improvement in empathy for the patient?
3. What background factors, among medical students’ gender and interest of specialty, were most strongly associated with improvements in empathy for patients?

## Method

### Study design

This was a single group pretest-posttest comparative study.

### Setting and participants

This study was carried out among fourth-year medical students at the University of Tokyo School of Medicine in Japan in the academic year (AY) 2018–19. There were two main reasons for targeting fourth-year students. First, previous studies in Japan reported that fourth-year medical students had the lowest empathy scores compared to other grades [13]. Second, fourth-year medical students are preparing for clinical practice the following year, and thus, the fourth year represents a time when students need to learn the magnitude of the psychological impact of medical professionals’ words on patients. We calculated the sample size of the current study with reference to previous studies on education that used interviews with patients [21]. Based on a set effect size of 0.2 and a significance level of 0.05%, the required number of participants was calculated at 199. Because the medical school where this study was conducted had approximately 100 students per academic year, the same questionnaire was administered to the same classes over two academic years to ensure the necessary number of students for the sample size. Medical students in their fourth grade in AY 2018 and AY 2019 were invited to classes to listen to patient storytelling. The inclusion criteria were that the participants had to attend the class from beginning to end. Those who could not respond to the questionnaire by the time the lecture started owing to tardiness were excluded from the research.

### Study instrument

#### Empathy for patients

The Japanese version of the Jefferson Scale of Empathy-Student Version (JSE-S) was used in this study. The scale has also been tested for reliability and validity [12, 30, 32]. JSE-S was relied on for the definition of empathy in the context of patient care as predominantly a cognitive attribute that involves the understanding of a patient’s experiences, concerns, and perspectives and a capacity to communicate this understanding with an intention to help. The scale includes 20 items, which were answered on a 7-point Likert-type scale (Strongly Agree = 7, Strongly Disagree = 1). The possible range of scores is 20–140; the higher the score, the more orientation toward empathic engagement in patient care.

#### Demographic and background factors

Gender, age, and interest of specialty were also obtained through the self-reported questionnaire. The classification of interest of specialty was made using the responses to the questions on career aspirations after graduation. Following previous studies, we asked them to choose between clinical medicine and basic medicine or whether they would be undecided about their career preference after graduation. In the analyses, respondents were divided into those who answered “basic medicine” (=1); “clinical medicine”; and “undecided” (=0). To compare between the participants interested in “people-oriented specialty” and “skills- or procedure-oriented specialties”, who have been shown to differ in their level of empathy toward patients, participants who were interested in “basic medicine” as their career were considered to correspond to the “technical−/procedure-oriented” in the previous study. As it is common in Japan for students to engage in clinical practice after graduating from medical school, we considered that those who wished to pursue basic research at the time of their fourth year of study had a strong inclination toward skills- or procedure-oriented specialties. We also recorded the academic year of the survey with reference to the participants’ response data (2018 = 0, 2019 = 1).

#### Study procedure

Classes on “Professionalism” in which the participants listened to patient storytelling were held on April 3, 2018, and April 4, 2019. The classes lasted for a total of 80 minutes (10 minutes of an introductory lecture, 30 minutes of patient storytelling, 20 minutes of group discussion among students, and 20 minutes of Q&A). The questionnaire survey was administered three times: at the beginning of the class (T1), immediately after the class (T2), and six months after the class (T3).

#### Exposure—a patient storytelling

We invited a patient storyteller who was diagnosed with chronic kidney disease in his elementary school age to the required course, “Professionalism,” offered to fourth-year medical students at the University of Tokyo School of Medicine. He gave a 30-minute talk under the title “The Power of Medical Professionals’ Words” about his experiences with his illness and his thoughts on the medical profession and answered questions from medical students for 20 minutes. The full text of his story is provided in Appendix 2.

The patient storyteller introduced three episodes related to the word cues suggested by doctors and nurses that marked turning points in his life. For each episode, the following sub-messages were conveyed: “Words of blame have the power to make patients sicker”; “Words of compassionate care have the power to strengthen a patient’s heart”; “Words of consistent support have the power to transform the patient from a sick state to an independent state as a human being.”

#### Data and statistical procedures

To examine RQ1, pretest-posttest comparisons were made in the combined data for AY 2018 and AY 2019 by using a t-test. Pretest-and posttest comparisons were also made for the AY 2018 and AY 2019 groups, respectively, and further examined for statistically significant differences between the two groups. We calculated Cohen’s *d* using the mean and standard deviation of the JSE-S scores in T1 and T2. To examine RQ2, analyses of variance for repeated measures were performed for the combined data for AY 2018 and AY 2019, and AY 2018 and for AY 2019 data alone, respectively. To examine RQ3, multiple regression analyses were performed using the JSE-S changes in T1-T2 (mean JSE-S score at T2 minus mean JSE-S score at T1) and T1-T3 (mean JSE-S score at T3 minus mean JSE-S score at T1). Independent variables were gender, interest of specialty, academic year, and the baseline scores of JSE-S. Age was excluded from the explanatory variables after confirming that there was no significant variation in the age of the participants in this study as Japanese medical students typically begin school immediately after graduating from high school. Cases with missing values were excluded from the respective analyses.

#### Ethical consideration

During the distribution of the questionnaire, students were reminded that their responses to the questionnaire would be voluntary; their individual responses would be kept strictly confidential and would not become part of their academic records. Written informed consent was obtained from the participants prior to the study; at the beginning of the class listening to the patients’ narratives and before the administration of the 6-month post-test. Withdrawal of consent was possible at any time by contacting YK by e-mail. The Institutional Review Board of the University of Tokyo (Tokyo, Japan) reviewed all methods of the current study for compliance with the “Ethical Guidelines for Life Sciences and Medical Research Involving Human Subjects” and approved the study (ethics review number 11860).

## Results

### Participant characteristics and baseline JSE-S scores

Of the total students in the classes (*n* = 202: *n* = 102 in the AY 2018 group and *n* = 100 in the AY 2019 group), 159 (78.7%) completed the JSE-S before and immediately after the class (T1 and T2). The following is the breakdown of JSE-S completion rates for the follow up survey (T3): 107, 67.3% (*n* = 44 (68.5%) in the AY 2018 group and *n* = 63 (68.5%) in the AY 2019 group. Of the 102 students who took the “Professionalism” classes in 2018, a total of 35 were excluded: those who did not respond (20 who left without submitting a survey), those who did not consent to the study (12 who submitted a survey but did not check consent to participate in the study), and 3 who did not complete the JSE-S before the class because they were late. Table 1 shows the participants’ characteristics and their baseline JSE-S scores. Despite the differences in response rates between the AY 2018 and 2019 groups, there were no statistically significant differences in gender ratio, percentage of clinical aspirations, age, or baseline JSE-S scores.

**Table 1:** Participants’ characteristics and baseline score of JSE-S

Variables	AY 2018		AY 2019		*p*	Total of AY 2018 + AY 2019				*p*
	N	(%)	N	(%)		N	(%)	JSE-S	(SD)	
Sex					0.127 ^a^					0.060^b^
Male	54	(80.6)	73	(79.3)		127	(79.9)	107.0	(11.4)	
Female	13	(19.4)	19	(20.9)		32	(20.1)	111.6	(12.2)	
Interest of specialty					0.562 ^a^					0.122^b^
Clinical medicine	40	(59.7)	60	(65.2)		100	(62.9)	109.3	(11.6)	
Basic medicine	8	(11.9)	11	(12.0)		19	(11.9)	106.0	(9.0)	
Not decided	19	(28.4)	21	(22.8)		40	(25.6)	105.2	(12.5)	
Age mean (SD)	21.5	(0.8)	21.3	(0.9)	0.122 ^b^	21.4	(0.9)	107.9	(11.7)	
JSE-S mean (SD)	109.3	(10.8)	106.9	(12.2)	0.197 ^b^			107.9	(11.7)	

JSE-S The Jefferson scale of empathy

^a^chi-square test

^b^Student’s t-test

### Changes in JSE-S scores immediately after listening to patient storytelling

The means and standard deviation of empathy pretest-posttest scores and summary results of statistical analyses are reported in Table 2. As shown in the table, an average increase of 3.3 scale points was observed in the empathy scores of the combined data for the AY 2018 and the AY 2019 groups, which was statistically significant (*p* < 0.001) (effect size = 0.4). Furthermore, both the AY 2018 and the AY 2019 groups showed statistically significant improvements, respectively. There was no statistically significant difference in the magnitude of change between the AY 2018 group and the AY 2019 group (*t*_(157)_ = 0.16, *p* = 0.86).

**Table 2:** Means and standard deviations (SD) of scores on JSE-S for total, AY 2018, group, and AY 2019 group before and after the educational intervention of patient storytelling

		T1 (pre-test)			T2 (post-test)					95% CL
Groups	N	Mean	(SD)	Mean	(SD)	Difference	t-test	LL	UL	Effect size
Total	159	107.9	(11.7)	111.2	(11.3)	3.3	5.2 **	2.1	4.6	0.4
AY 2018	67	109.3	(10.8)	112.7	(10.3)	3.4	4.3 **	1.9	5.1	0.5
AY 2019	92	106.9	(12.2)	110.2	(11.9)	3.3	3.5 *	1.4	5.1	0.4

*CL* Confidence interval, *LL* Lower limit, *UL* Upper limit

Effect size = Cohen’s *d*

*p < 0.05, **p < 0.01

### JSE-S scores after six months

**Fig. 1:**
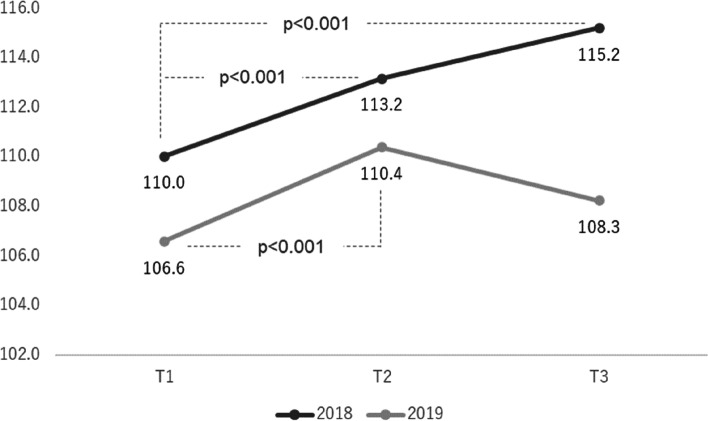
Changes in mean scores of JSE-S in different times in AY 2018 group and AY 2019 group

Means, standard deviations, and summary statistical results of repeated measure analysis of variance comparing the three points of surveys are reported in Table 3. As shown in the table, for the combined data, the JSE-S score of T3 measured six months after T1 was higher than that of T1 and was also statistically significant. As shown in Fig. 1, for the AY 2019 group, significant improvements that were observed in empathy scores from T1 to T2 dissipated at T3 (T1 < T2 = T3, *p* = 0.01). On the other hand, the AY 2018 group scored higher at T3 than at T1 and T2 (T1 < T2 < T3, *p* = 0.01).

**Table 3:** Change in mean scores of JSE-S in different times in AY 2018 group and AY 2019 group

		Administration of JSE-S							
		T1 (pre-test)		T2 (post-test)		T3 (6 months later)			
Groups	N	Mean	(SD)	Mean	(SD)	Mean	(SD)	*F*-ratio	Differences
Total	107	108.0	(11.8)	111.6	(10.9)	111.1	(11.9)	7.7 **	T1 < T2 = T3
AY 2018	44	110.0	(10.3)	113.2	(9.7)	115.2	(10.5)	5.9 *	T1 < T2 < T3
AY 2019	63	106.6	(12.7)	110.4	(11.5)	108.3	(12.0)	4.5 *	T1 < T2 = T3

Analysis of variance was used for repeated measure design.

*p < 0.05, **p < 0.01

### Background factors associated with changes in JSE-S

Table 4. shows the results of multiple regression analysis using the change in JSE-S scores from T1-T2 and T1-T3 as the objective variable. Three cases were excluded in the analysis with the change in JSE-S scores between T1 and T2 as the dependent variable and two cases were excluded in the analysis with the change in JSE-S scores between T1 and T3 as the dependent variable owing to missing responses. Statistically significant associations were found for the interest of specialty (*β* = −0.15, *p* = 0.032) and baseline JSE-S scores (β = −0.39, *p* < 0.001) with improved empathy scores between T1 and T2. Gender showed no significant association. The academic year was not significantly associated with a change in T1-T2 scores (β = −0.05, *p* = 0.48) but with a change in T1-T3 scores (β = −0.23, *p* = 0.01). No interaction effect between academic year and other variables was found.

**Table 4:** Multiple regression analysis with the amount of change in scores of JSE-S﻿ (T1-T2 and T1-T3) as objective variables

**Variables**	**T1-T2** (*N* = 156)			**T1-T3** (*N* = 105)		
		**95% CL**			**95% CL**	
	**β**	**LL**	**UL**	**β**	**LL**	**UL**
Gender	0.00	−2.96	2.92	−0.02	−5.26	4.22
Clinical orientation	−0.15*	−7.52	−0.08	−0.04	−7.89	4.98
Baseline scores of JSE-S	−0.39***	−0.37	−0.17	−0.48***	−0.63	−0.29
ΔR^2^	0.17			0.21		

Explanatory Variables: gender (male = 0, female = 1); clinical orientation (aspiring clinical medicine/Not decided = 0, aspiring basic medical research = 1); JSE-S(T1) = baseline score of the Jefferson scale of empathy

*CL* Confidence interval, *LL* Lower limit, *UL* Upper limit

*p < 0.05, **p < 0.01, *** *p* < 0.001

## Discussion

The participants’ JSE-S scores improved significantly immediately after listening to patient storytelling and remained in the improved state six months after the class. The magnitude of improvement in JSE-S scores for the participants of this study (3.3 points, effect size 0.4) was higher than those in previous studies that used patient narratives—education using medical dramas (effect size 0.18) [15], education using video interviews with pediatric patients with hereditary diseases and their families (effect size 0.14) [21], and an educational program in which teachers recited patients’ stories (score change 0.9 scale points) [24]. Since this study did not have a control group, the increase in scores cannot be attributed to the effect of listening to the patient storytelling. However, previous studies reported that JSE-S scores of the control group during one lecture did not change or slightly increased by less than one point [23, 33]. The mid-to long-term change in JSE-S scores was also lower than the baseline score after 10 weeks in the U.S. [23]. Given these considerations, it can be inferred that the maintenance or increase in JSE-S scores over a six-month period in the participants of this study differed from natural score changes. The results may suggest that when conducting future studies with a control group, a medium-term follow-up period of about six months may provide an insight into the extent to which educational effects are maintained.

A unique finding of the current study was that long-term changes in JSE-S scores differed between the AY 2018 and 2019 groups. The AY 2019 group’s score, which dropped slightly immediately after the class but remained higher than before the class, may indicate that the educational effects of this study were moderately maintained. This trend is consistent with changes in general educational intervention effects. Consequently, continued and repetitive education is needed. In contrast, the scores for the AY 2018 group six months after the class were even higher than those recorded immediately after the class. This trend of change in scores was similar to the improvement seen in a study on an educational program with reinforced education 10 weeks after the initial education [23]. Therefore, it is undeniable that during the follow-up period, only the 2018 group may have had some event that corresponded to the enhanced education. There was no difference in the annual educational curriculum received by the AY 2018 and 2019 groups, but the education received prior to the T3 test was different. Specifically, the 2018 group received a “clinical practice orientation” on the attitudes required for a student physician, while the AY 2019 group received a “clinical reasoning lecture” prior to the T3 surveys. It is possible that the clinical practice orientation had an impact on the students’ JSE-S scores that were comparable to the reinforced education mentioned in previous studies.

There was a small but significant association between participants’ interest of specialty and the change in JSE-S scores immediately after listening to the patient storytelling. This indicated that being technology-oriented was significantly associated with improved empathy scores. Previous studies reported that medical students who aspired to gain a “people-oriented” specialty after graduation from medical school obtained a significantly higher JSE mean score than others who were interested in “skills- or procedure-oriented” specialties [14–16]. This may be explained by the learning effects of empathy through narratives based on adult learning theory. Since narratives help listeners relive the experiences and feelings of the patient storyteller [18, 20, 28, 29], patient storytelling might promote perspective taking—understanding something from another person’s perspective—regardless of the participants’ interest of specialty. The results of the current study may indicate the usefulness of patient storytelling as an empathetic education for “skill/procedure-oriented” medical students.

Although many previous studies have noted that gender would be a differential factor in the educational effects of empathy [11, 12, 34, 35], this study found no significant association with change in JSE-S scores immediately and six months after the education. This may be explained by the small proportion of women in this study. Though the percentage of female medical students in Japanese medical schools has been increasing in recent years, the ratio of male to female students remains the same. However, the male to female ratio of the medical school where this study was conducted was approximately 7 to 3. Therefore, the number of female students was small, and gender differences in educational effects may not have been adequately examined.

There are several limitations to this study. First, the study was designed without a control group. Second, only medical students from one university were included in the study. Therefore, future studies should be designed with a control group to examine educational effects in a multicenter setting. Third, considering that the respondents’ rate was low (T2: 78.7% and T3: 67.3%), the results of the analysis may have been biased toward a more desirable change in scores because the analysis was biased only toward the responses of medical students who cooperated with the survey. In future studies, it would be useful to carefully examine the results by adding qualitative analysis, such as students’ impressions, which cannot be captured by changes in scores alone. Fourth, this study used only one patient storyteller. Future studies are required to examine whether storytelling by other patients with different diseases and different life backgrounds would have the same effect. Despite the above limitations, this is the first study to clarify the educational effects of empathy through patient storytelling using a quantitative method, and the results may provide a first step toward future research.

## Conclusion

This study reported a statistically significant increase in medical students’ empathy for patients in a Japanese medical school immediately after hearing patient storytelling, which they may have maintained six months later. Further, the study examined medical students’ background factors associated with improved empathy for patients and reported that clinical orientation was more significantly associated than gender. Our findings may suggest that patient storytelling would be useful for cultivating empathy for patients among undergraduate medical students. Patient storytelling would be useful in humanistic and professional education curricula to foster empathy for patients among medical students. The involvement of patients and citizens in medical education is expected to be promoted. It is to be expected that more medical schools will use patient storytelling to educate medical students in humanistic and communication education.

## Supplementary Information

**Additional file 1.** Manuscript of patient storytelling used in the current study.

## Data Availability

The datasets used and/or analyzed during the current study are available from the corresponding author on reasonable request.

## Abbreviations

AY: Academic year
JSE-S: Jefferson Scale of Empathy-Student Version
SD: Standard deviation
