# Supplementary material for: Using patient storytelling to improve medical students’ empathy in Japan: a pre-post study
Source: BMC Med Educ. 2023 Jan 27;23:67. doi: 10.1186/s12909-023-04054-1 (PMC9881337; doi:10.1186/s12909-023-04054-1)
Supplement: Supplementary file 1 — Additional file 1. Manuscript of patient storytelling used in the current study. [file 12909_2023_4054_MOESM1_ESM.docx]

**Appendix -** Manuscript of patient storytelling used in the current study

**Title: "The Power of Medical Professionals' Words”**

**Greetings**

Good morning, my name is Junya Tanaka, and I am a patient speaker. First, I would like to express my gratitude to Dr. Sun of the University of Tokyo for providing me with this opportunity. Thank you very much. The theme of my talk this time is "The Power of Medical Words". I am a patient as well as a medical professional. Today, I would like to discuss with you communication between medical professionals and patients from the perspective of patients, based on my experiences. I look forward to working with you.

**Introduction**

Now, can you guys solve this problem? Find the area of the triangle. I am sure you all can figure it out right away. The correct answer is 12 square meters. It is easy, isn't it? If you apply the formula for finding the area of a triangle, you can immediately get the correct answer, and anyone can see that it is definitely 12 square meters. Now, consider the following problem. These are the words that patients and their families ask in desperation: "Doctor, please help me;" "Doctor, please make my disease go away, I beg you..." Now, what words will you tell them?

There is probably no correct answer to this question, nor a formula like the one we have just described that will lead us to the correct answer. I would like to share my experience with you, and we will discuss together what physicians can share with patients and their families.

**Self-Introduction**

I am currently 40 years old. I was born in Wakayama Prefecture and currently live in Osaka Prefecture. I had chronic kidney disease and had been on dialysis for 23 years. Three years ago in 2015, I was able to receive a donated kidney transplant and am no longer on dialysis. I am a nurse, a certified chronic disease nurse specialist, and I support along with people from various professions people with chronic diseases, and their families. I don't think I need to explain details about chronic kidney disease to you, the medical students. I had a disease called reflux nephropathy, which led to kidney failure. I had undergone hemodialysis and kidney transplantation.

The main message I would like to share with you this time is that the words of a medical professional can make a patient a sicker person and a human being.

**Episode 1**

This is a picture of mine shortly after I was born [He shows a photograph]. I have a brother two years older than me. Sometime after he was born, tests showed that there was protein in his pee. However, when I was born, they did not have me specifically tested—because my birth occurred 40 years ago in a rural hospital. But my mother became concerned and did a simple test on my pee at home. And my urine showed protein. She became concerned and immediately went to the hospital. The doctor told her, "there is nothing to worry about. Don't worry, mother. It will get better as the baby gets older. After that, I was raised without any treatment.

This picture was taken when I entered elementary school [He shows a photograph]. Even after entering school, I always continued to have urinary protein at school checkups, so I regularly visited a nearby hospital. At my school, children could join either the Little League baseball or soccer team in the second grade. I was a big fan of the Yomiuri Giants professional baseball team and wanted to join Little League. I told my mother I wanted to play baseball, but she said, "Oh, no". I begged her repeatedly and told her I would study hard and eat broccoli that I hated; however, she did not budge. Then I asked the doctor at the hospital if I could play baseball. The doctor looked a little troubled and said, "I don't think it's possible for you right now. But you will be able to play when you get older. Then he added, "Your disease will be cured when you grow up." "If you are a good boy, you will get better." So, I decided not to say anything that would embarrass my mother, and I never talked about Little League again. Despite my regular visits to the hospital, I was not aware that I was ill because I did not experience pain anywhere in my body.

The turning point was when I was 10 years old. When I was watching TV at home, the ceiling started spinning in circles, and it did not go away even when I closed my eyes. My mother was so worried that she rushed me to the hospital for a checkup and found that the amount of protein in my urine was three times more than usual. My doctor told me, "We can't do any more tests or treatment here. You should go to a big hospital in the city," and I was admitted to a university hospital.

This picture shows me during that time of my hospitalization. As you can see, I have gained a lot of weight. My parents thought I was just fat because I was eating a lot, but in hindsight, my kidneys had deteriorated and I had edema, a condition called nephrosis. Then I underwent a kidney biopsy at a university hospital. Seeing the results, the doctor told my parents, "What have you been doing? In this condition, he will end up on dialysis." Only when I was much older, I was told that the doctor spoke to my parents about the results of my kidney biopsy in this way. And based on the results of the biopsy, two restrictions were imposed. The first was dietary restrictions; the doctor instructed me to take 30 grams of protein and 5.0 grams of salt or less. Can you guys imagine what kind of diet that was? The second was exercise restrictions. All physical education at school was banned. I am sure you all realize that these instructions were not correct. The current guidelines advise against such an instruction. However, 30 years ago, lifestyle restrictions recommended in the guidelines were different. Since then, evidence has been produced based on the results of various studies, and the current guidelines have been established. However, my family and I have followed the doctor's instructions honestly all the way up until the start of my dialysis.

And during hospitalization, I was hearing a new word: "dialysis treatment.” I heard it from my mother. At that time, she said in tears, "Let's do our best not to be on dialysis.” I had no idea what "dialysis" meant. At that time, there was no Internet or smartphones like now, so I could not search for the word quickly. So, I looked it up in Japanese dictionaries and family medical books. Then, I found a lot of difficult words that a 10-year-old could not understand. Though I did not understand it, I thought that dialysis was a scary thing—If I went on dialysis, I might not be human anymore. I remember I could not sleep under the covers because of fear.

Perhaps the doctor was medically correct when he said, "In this condition, you will end up on dialysis." However, I could not feel the future in the doctor's words. As for my parents, they felt they were being blamed and accused, and these words stung like a knife. And hospitalization was the beginning of recuperation for me and my family. What I would like to convey to you from this episode is that “Words of blame have the power to make patients sicker.”

**Episode 2**

Now, here is a picture taken about a year after I was discharged from the hospital [He shows the picture].

When I was in the hospital, I had a full head of hair, and I did not like the way everyone touched my head, so I let my hair grow long after leaving the hospital. Because I strictly followed the dietary restrictions, the swelling went away, and one year after leaving the hospital, I lost more than 10 kg of weight.

Now, I would like to introduce my life after leaving the hospital. First, my school provided the lunch; however, because of my dietary restrictions, my mother prepared a lunch box for me every day. The lunch was mainly vegetable-based and contained little salt. My mother asked the schoolteacher for a school lunch menu and made side dishes every day similar to the school lunch menu for that day. I think my mother was trying to prevent me from being teased or bullied by my friends because I was the only one who was not eating the school lunch.

The only trouble was that there was a vinegar dish in every lunch box. Every morning I put my lunchbox into my bag and walked to school with my friends. My friends and I would joke around and play with each other, swinging our lunch-bags at each other. And this caused the lunch box to turn over; when I opened the lunch box at lunchtime, I found the vinegar in the vinegar side dish had sunk into the rice, making it sushi rice.

One day, when the school lunch was curry, my mother prepared a lunch box with curry rice. My mother could have just separated the containers, but she did what she always did: she put the vinegared side dish next to the rice, wrapped the curry in a plastic wrap, and put them in the same container. Every morning I put my lunchbox into my bag and walked to school with my friends. My friends and I would joke around and play with each other, swinging our sub-bags at each other. At lunchtime, when I opened the lunch box, it was vinegar rice and curry. Today, a famous sushi chain in Japan is marketing the combination of sushi rice and curry as sushi curry. I think my mother had developed the dish 30 years ago.

Second, owing to my exercise restrictions, I could not participate in my school physical education classes and always merely watched the activities; I was not allowed to do even simple exercises. Children are honest. My friends said, "He's slacking off again," or "It's so unfair that he doesn't have to run the marathon.” I knew there was no point in talking back to them every time, so I just smiled at them.

Third, dozens of oral medications were started. Just opening my lunch box in the classroom was enough to draw attention because I was the only one doing something different from my classmates, but on top of that, it was extremely difficult to take different medications in the classroom. So, I always took my medicine in the bathroom. At first, I had a water bottle with me, but my friends teased me saying, "it is so unpleasant to bring a water bottle to the bathroom.” So, I was forced to take both powders and pills without water, which became my only special skill.

Eventually, my friends, who at first looked at me as if I was a rarity, gradually became distant or avoided me. It is not that I was bullied or ignored. I didn't say it out loud, but I could sense in their attitudes and gazes that they felt sorry for me because of my illness. Not only my friends but also schoolteachers and relatives also behaved in that manner. I heard people around me talking about me and even my mother behind my back, "It's too bad that such a small child is so sick," and " she should have given birth to a healthier baby." I felt all those words seeping through my skin and into my body.

I thought I was no different from everyone else—just eating lunch I brought from home instead of school lunch, watching gym class instead of participating in it, and taking my medicine. But owing to the attitudes of everyone around me, the idea that having a disease was something to feel sorry for and that I was different from others seemed to have been unconsciously imprinted on me. Of course, part of me was in denial, saying, "That's not true." But the more I denied and rebelled, the smaller and smaller I became.

This picture was taken when I was 15 years old [He shows the photograph]. I was a serious person, so I followed my diet and exercise restrictions to the letter. However, at the age of 15, I began to catch a cold every month. When I caught a cold, my kidneys would go bad very quickly. It was just three months before I was to take the high school entrance exam. I was hospitalized and underwent surgery in preparation for dialysis. And this was a month after I was discharged from the hospital. I still remember the morning of January 31. When I woke up, I could not move, and just moving a little made me nauseous. I went to the hospital immediately because my body was different from usual, and I was told that I should start dialysis right away, so I was hospitalized and started the dialysis treatment.

The dialysis needle is about as thick as the tip of a ballpoint pen, but I do not remember any pain at all. I don't know if I was nervous or what. I just looked at my arm where the needle was stuck and saw my blood flowing through various tubes and circling around in the machine. Then I remember feeling an indescribable kind of helplessness and defeat. I spent the four hours of treatment thinking, "I have worked so hard and so diligently, I have put up with sweets and juice, and yet...why me...?

After the treatment was over, I returned to my room. The room was private, and there was food on the bedside table. It was January, so it was already dark in the evening, but I did not turn on the light or the TV in my room. In my mind, I was filled with a sense of despair, a vague fear and anxiety about what would happen to me in the future, and a feeling of resignation that I did not really care anymore.

Then I heard a knock at the door. I wondered who it was, but I did not answer. Then the door was opened, and I heard a voice saying, "What are you doing without turning on the light, exam student? It was a familiar voice. It was a nurse from the dialysis room where I had been treated just a few minutes ago. I was surprised that he suddenly turned on the light and told me that I was an exam student. When I did not speak anything, he said, "You need to eat and study as soon as possible. You are going to take an exam soon." I was annoyed at the repeated use of the word "examinee" and at the nurse who was talking to me one-sidedly, ignoring my current feelings, and turned my back on him. Then the nurse asked me, "Why are you so depressed? What are you depressed about, you idiot? Do you think your life is over after starting dialysis, or do you think you are not normal? From my point of view, Tanaka-kun is just like any other 15-year-old kid around here. I'll be waiting for you tomorrow in the dialysis room. Then, eat your dinner and study," saying this, he left. He didn't even close the door. It was an event that lasted for only a couple of minutes. At first, I was very angry. But as time passed, I became happy about what the nurse said, "You are just like any other 15-year-old kid around here," repeating it over and over in my mind. And I realized that my heart was becoming lighter. This may be because his response was different from other expressions, such as "poor you" and "I'm sorry for your loss," sympathetic attitudes and stares from those around me, which unknowingly made me assume that I was poor or weak. It was the moment when I was freed from such a spell on myself and could think that I might be able to do anything even if I have a disease.

From the words of the nurse, I learned that “Words of compassionate care have the power to strengthen a patient's heart.”

**Episode 3**

Later, I successfully passed the high school entrance exam, went to nursing school, and became a nurse. Like any other profession, it was a challenge— in the first year of employment, there is a lot to learn, and one makes many mistakes. In my case, I made the same mistake twice before I learned from it, and my seniors would often get angry with me, saying, "You said that the last time, didn't you? You know what I mean? I underwent dialysis three times a week after work. Every day I went back and forth between work, home, and the dialysis clinic. I had been self-reliant since I was in nursing school, so I was good at cooking for myself. But I was too tired to cook when I got home. So, I spent many days eating only what I bought at convenience stores and supermarkets. However, since restaurants served salty food, I ended up drinking a lot of water. Thereafter, when I went to the dialysis room, the doctors and nurses would always tell me, "Why are you gaining so much weight, what on earth are you eating? First, I laughed bitterly, but I kept gaining weight again, and the days of bad data kept on coming. The doctors and nurses would say at the first word, "You're gaining again, you said the other day that you would do your best next time, what have you been doing?

I did not say anything in reply; I did not even look them in the eye; all I could say was, "I'm sorry, I'll do my best next time. Of course, in my heart, I didn't feel sorry, not even a millimeter. In my heart, I felt that no one understood me, and I was angry at all the medical staff. I hated coming to dialysis, I hated seeing their faces, and I hated myself for becoming so frustrated and despicable.

Then my workplace changed; so did the dialysis clinic. The nursing work was the same. I sometimes gained weight, as I still ate out a lot. But the doctor at the clinic would always look at the data showing my weight with a nod, come to my bedside, and say, "How are you doing at work, aren't you busy? Are you getting any rest? Before that, the doctors and nurses at the clinic only asked me about my weight and data figures, but this doctor was totally different. He would slowly ask me about my work and how I was spending my days off from the same perspective. First, I was wary of him because I thought she was a strange teacher, but then I started talking to him about myself. I told him that I was too busy to cook meals even if I wanted to, that I knew full well that my data was bad, and that I hated myself the most for such a lack of self-control. Then, smiling, he said

"I see. But, well. There are good days and bad days. You just must do your best till when you feel like you can. But if your data are bad or you gain a lot of weight, I am worried about your condition. That's the same for me and all the nurses here. Just remember that. Hearing those words, I became aware of the existence of medical personnel who were concerned about me. It also made me realize that I should try my best to manage my diet regimen, stop being mean and stubborn, and stop giving up on myself for not being able to do it.

From this doctor's words and unspoken attitude and behavior, I learned that words of a medical practitioner who tries to be close to the patient, whether or not the patient's self-management is going well, has the power to transform the patient from a sick state to an independent state as a human being.

**Conclusion**

In my life with chronic kidney disease, I have learned from the words of a doctor at a university hospital, "Words that do not give hope make a sick person sicker." From the words of a nurse I learned, "Words can make a sick person stronger." And the words and behavior of a doctor at a dialysis clinic taught me that, "Words has the power to transform the patient from a sick state to an independent state as a human being.” The message I want to convey to you is that “Words of consistent support have the power to return a sick person to a dignified human being.”

Finally, you will become doctors in the future. What words will you continue to say to those who are ill?

Thank you.
